# Non-Employment Histories of Middle-Aged Men and Women Who Died from Alcohol-Related Causes: A Longitudinal Retrospective Study

**DOI:** 10.1371/journal.pone.0098620

**Published:** 2014-05-29

**Authors:** Tapio Paljärvi, Pekka Martikainen, Taina Leinonen, Tiina Pensola, Pia Mäkelä

**Affiliations:** 1 Alcohol and Drugs Unit, National Institute for Health and Welfare, Helsinki, Finland; 2 Population Research Unit, University of Helsinki, Helsinki, Finland; 3 Health and Work Ability, Finnish Institute of Occupational Health, Helsinki, Finland; Universität Bochum, Germany

## Abstract

**Background:**

Long-term patterning of non-employment among problem drinkers is poorly understood. We determined the level and timing of non-employment, and the relative contribution of various types of non-employment among middle-aged persons who died of alcohol-related causes.

**Methods:**

We conducted a longitudinal retrospective register-based study of Finnish men and women aged 45–64 years who died of alcohol-related causes (n = 15 552) or other causes (n = 39 166) in the period 2000–07, or who survived (n = 204 422) until the end of 2007. We traced back the number of days in employment and non-employment for up to 17 years before death or before the end of the study period for the survivors.

**Results:**

The majority (≥56%) of persons who died of alcohol-related causes were in employment up to ten years before death. Over the 17-year period before death, those who died of alcohol-related causes were in employment on average two years less (mean 6.3 years, 95%CI 6.2–6.4) than those dying of other causes (8.2, 8.1–8.3), and five years less than survivors (11.6, 11.5–11.7), when sex and age were adjusted for. The relative role of various types of non-employment differed markedly across the two mortality groups. Among those who died of alcohol-related causes, unemployment accounted for 54% of the total burden of non-employment, in comparison with 29% among those who died of other causes. In contrast, disability pension accounted for 41% of the total burden of non-employment among those who died of alcohol-related causes, but 65% among those who died of other causes.

**Conclusions:**

The results indicate the feasibility of preventing movement out of employment among middle-aged men and women with severe alcohol-related harm, provided that they are identified early on during their working careers and offered effective interventions.

## Introduction

Labour force non-participation has become highly relevant to public policy because governments try to address budget deficits by cutting costs of welfare systems, and because projected changes in population structures in many Western countries have prompted concerns over long-term sustainability of public finances.[1], [2] One potential solution to these financial demands is to increase the employment rate by tackling causes of labour force non-participation.

Due to the well-established acute and chronic adverse effects of excessive alcohol use,[3], [4] problem drinking impacts both the overall probability of becoming employed and the mechanisms of temporary and permanent movement out of labour force.[5]–[14] Overall, the effect of problem drinking on the probability of being in employment is likely to be time-dependent, particularly among those with a less severe condition: when unemployment rate is low, especially mild forms of problem drinking may have little adverse effect on employment, but when the unemployment rate increases, problem drinkers may be more severely affected.[11] It has been claimed that the labour force non-participation of problem drinkers is not only a burden to the benefits system,[15] but also that at an individual level, being in employment facilitates successful recovery from alcohol misuse, and therefore interventions should support problem drinkers to gain employment.[16]

However, it is unclear to what extent problem drinkers are employable in terms of their long-term functional capability for work.[17] In addition to potentially incapacitating health problems, severe forms of problem drinking frequently co-occur with various aspects of social dysfunction likely affecting the ability to gain and maintain employment.[18]–[20] For developing effective interventions promoting sustained employment among problem drinkers, we need information on how labour market disadvantage is accumulated over the life course of these persons. By establishing the long-term employment histories of problem drinkers we can assess to what extent there is potential to prevent movement out of employment among them that could be capitalized on by effective interventions. For example, if problem drinkers are outside the labor force for the most part of their life, their employability will be low and interventions less effective due to persistent marginalization. Furthermore, information on the timing of different types of non-employment could be used to identify windows of opportunity during which interventions aimed to prevent alcohol-related premature exit from the labour force should be implemented.

The long-term patterning of non-employment among problem drinkers is poorly understood mainly because of lack of suitable data. Therefore, the purpose of this register-based study is to retrospectively examine the non-employment histories of middle-aged men and women who died from alcohol-related causes. Comparisons with survivors (general population) and those who died from other than alcohol-related causes enable us to make conclusions on the special characteristics of the employment trajectories of problem drinkers who are at risk of experiencing severe alcohol-related health outcomes. Survivors represent the normative general population employment trajectory and those who died from other than alcohol-related causes represent the general employment trajectory related to poor health in the years before death. Using this novel design we established the average level and timing of non-employment before alcohol-related death, and the relative contributions of unemployment, medically certified sick leave, and disability pension to the total burden of non-employment. To our knowledge our study is the first to establish the long-term patterning and the relative contribution of various types of non-employment in the life course of problem drinkers at risk of experiencing severe alcohol-related health outcomes.

## Materials and Methods

### Ethics statement

The sampling and data linkage was approved by the ethics committee of Statistics Finland.

### Data

The register-based study data consists of an 11% random sample of the population living in Finland during the period of 1987 to 2007. Because our focus was in the employment histories of persons who died from alcohol-related causes (a proxy for a history of problem drinking), we collected an additional sample of persons who died in 2000–07 in order to increase precision. This oversample of deaths enabled us to capture 80% of all deaths that occurred in Finland during the study period, which was the maximum representation of deaths retrievable from the records of Statistics Finland due to data protection rules. In addition, information from the nation-wide registers of Statistics Finland (employment, unemployment, sociodemographic factors, and causes of death), Social Insurance Institution of Finland (medically certified sick leaves and disability pensions), and Finnish Centre for Pensions (disability pensions) were linked to the data. Statistics Finland carried out the sampling and data linkage using the unique personal identity code issued to all Finnish residents, which was available in all registers used in this study.

**Figure 1:**
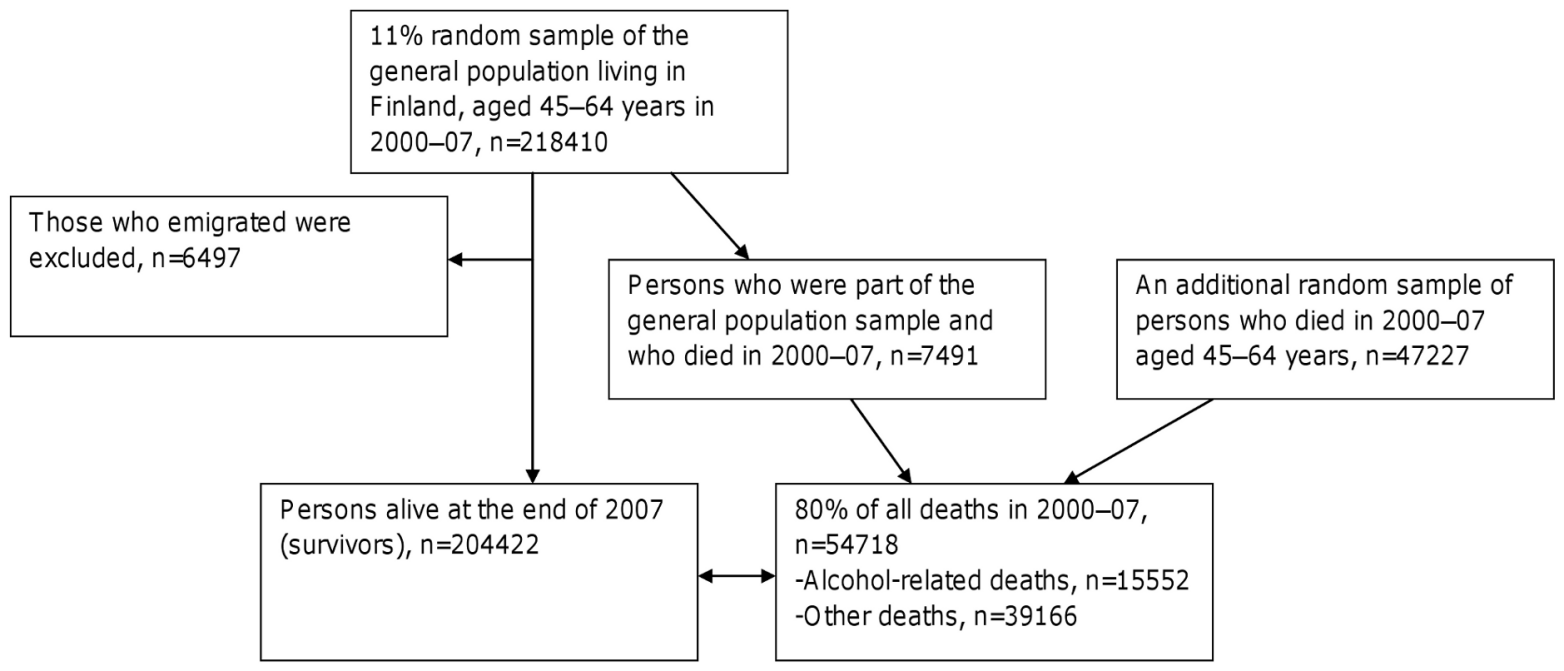
Flow chart of the study population.

Men and women aged 45–64 years at the time of death were eligible for the present analyses because in this age group alcohol-related mortality is at its highest and the employment histories are at their longest. Survivors were of the same age group as the deceased and were alive at the end of 2007. Employment histories were traced back for up to 17 years before death or the end of study period. The study data consisted of 204422 persons who survived until the end of 2007 and 54718 persons who died in 2000–07 (figure 1). Of the deceased, 15552 died from alcohol-related causes and 39166 died without alcohol involvement (other deaths). Persons who emigrated during the study period were excluded (<4%) from the data.

Causes of death were identified using the International Classification of Diseases, tenth revision (ICD–10) codes. In identifying alcohol-related causes we used the following codes F10, mental and behavioural disorders due to use of alcohol; G312, degeneration of nervous system due to alcohol; G4051, epileptic seizures related to alcohol; G621, alcoholic polyneuropathy; G721, alcoholic myopathy; I426, alcoholic cardiomyopathy; E244, alcohol-induced pseudo-Cushing syndrome; K292, alcoholic gastritis; K70, alcoholic liver disease; K852, alcohol-induced acute pancreatitis; T51, toxic effects of alcohol; X45, accidental poisoning by and exposure to alcohol, X65, intentional self-poisoning by and exposure to alcohol; Y15, poisoning by and exposure to alcohol, undetermined intent). In order to improve the coverage of identifying problem drinkers, we used information on recorded underlying and contributory causes of death. Using also contributory causes of death has been shown to improve the coverage of the role of alcohol in mortality.[21] The most important groups that will be identified by using contributory causes are persons who died from external causes and cardiovascular diseases with alcohol intoxication as a contributory cause, and persons who died from various diseases with alcohol dependence as a contributing factor. In other words, persons defined as problem drinkers were either harmful or dependent drinkers at the time of death.

For each calendar year, time in employment and unemployment was recorded as months and converted to days. Time on medically certified sick leave was recorded as number of days. For sick leaves we had information on those periods eligible for sickness allowance from the Social Insurance Institution of Finland, i.e. medically certified sick leave periods lasting over 10 working days (long spells). For disability pensions, end-of-year information was used to determine disability pension status. Those who were on disability pension at the end of each year were assigned to have 365 days of disability pension during that year. Total non-employment was calculated as the sum of days of unemployment, medically certified sick leave, and disability pension.

Information on socioeconomic status was recorded in five-year intervals, and to ensure comparability, we used the most recent information that was available for all year-of-death groups, which was socioeconomic status in 1995; categorized as 1) upper and lower white collar workers, 2) manual workers, and 3) others. Similarly, for marital status we used the most recent information available for all subjects, which was marital status in 1999; dichotomized as 1) married and 2) non-married (single, divorced, or widowed).

### Statistical analyses

For those who died of alcohol-related causes and other causes of death in year y_i_ (_i_ = 2000–07), time before death was calculated in years. In order to avoid confounding by period effects, the comparison group of survivors was formed separately for each year y_i_ by randomly assigning each survivor to one of the eight years.

By using generalized estimating equations (GEE) we estimated the group-level mean trajectory over all individual trajectories. This method adjusts the standard errors for the within-subject clustering of data over repeated measurements. We used an autoregressive correlation structure on the assumption that within-subject measurements closer in time are more highly correlated than measurements further apart. The results are presented as estimated marginal means with their 95% confidence intervals adjusted for sex and age, and plotted against time before death for the deceased or time before end of follow-up for survivors. Analysis weight variable was used in all the analyses to correct for the different sampling probability between survivors (11%) and the deceased (80%). SAS/STAT software's GENMOD procedure was used to perform the GEE analyses.

## Results

**Figure 2:**
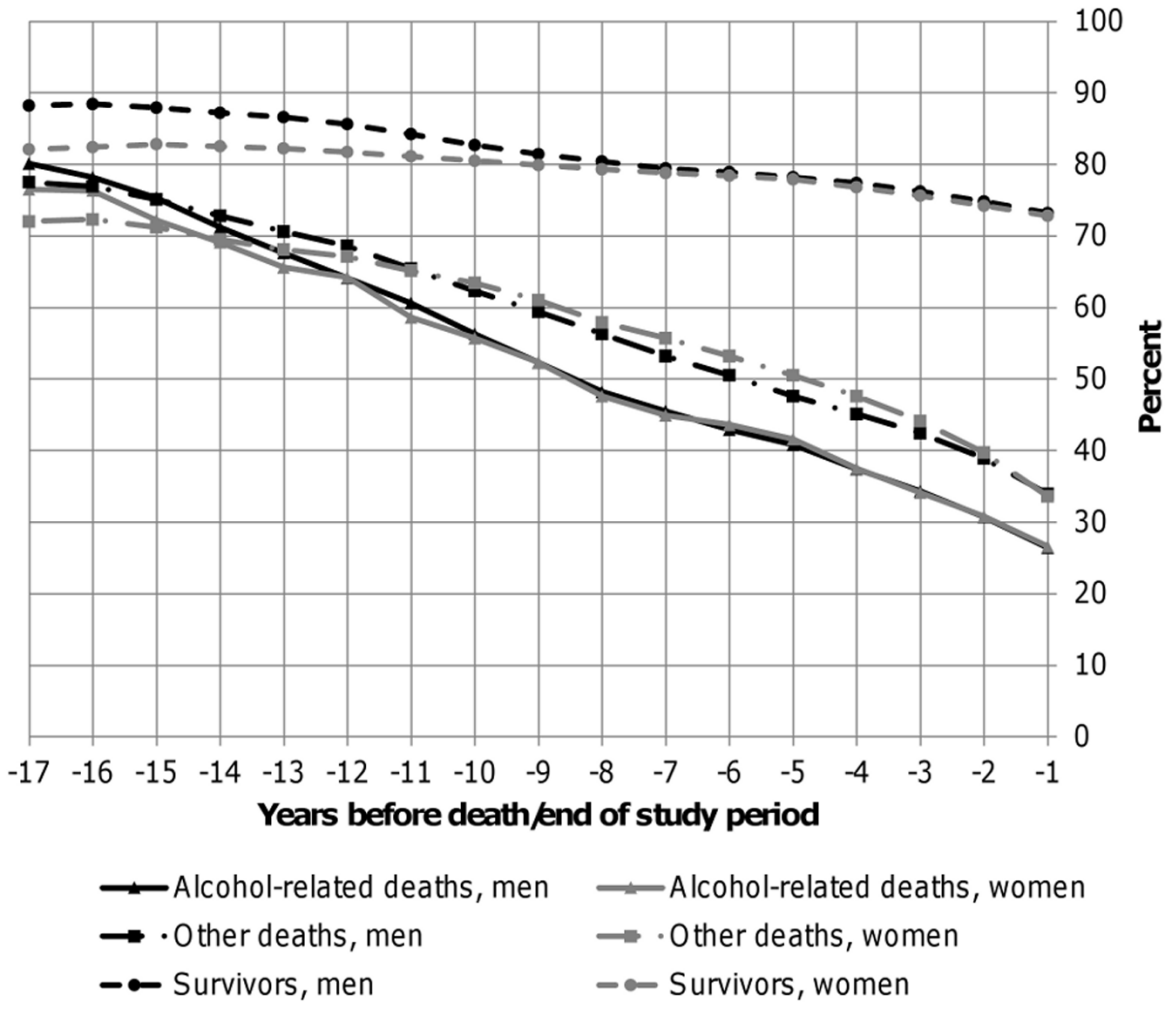
Proportion of men and women in employment at a given year before death/end of study period.

Of those who died from alcohol-related causes a larger proportion were men 12694/15 552 (82%), compared to those who died of other causes 25262/39 160 (65%). Furthermore, of those who died of alcohol-related causes a larger proportion were manual workers and non-married, compared to those who died from other causes and survivors, both in men and women (table 1). Of those who died of alcohol-related causes, 26% were in employment at one year before death, 41% at the fifth year before death, 56% at the tenth year, and 79% were in employment at the 17^th^ year before death (figure 2). Thus, at the tenth year before death, the majority (56%) of those who died of alcohol-related causes were still in employment. In comparison, at the tenth year the proportion of persons in employment was only slightly higher among those who died of other causes (63%), but markedly higher among survivors (82%).

**Table 1:** Characteristics of the study population.

	Men (n = 137980)			Women (n = 120116)		
	Alcohol-related death	Other death	Survivor	Alcohol-related death	Other death	Survivor
No. of subjects^a^	12 694	25 262	100 024	2 858	13 898	103 360
Mean age in years at death/end of study period (SD)	55 (2.8)	57 (2.7)	54 (7.7)	55 (2.8)	57 (2.7)	54 (7.8)
Manual workers^b^	8013 (63%)	13 662 (54%)	44 309 (45%)	1191 (42%)	4697 (34%)	26 596 (26%)
Non-married^c^	8818 (69%)	12 772 (51%)	35 264 (35%)	1732 (61%)	6555 (47%)	36 850 (36%)

a Unweighted, ^b^year 1995 status, ^c^year 1999 status.

The average sex and age adjusted annual number of days of employment was lower among persons dying of alcohol-related causes than among survivors, or among those dying of other causes (table 2). In terms of years of employment accumulated over the 17-year period, those who died of alcohol-related causes were in employment on average two years less (mean 6.3 years, 95%CI 6.2–6.4) than those dying of other causes (8.2, 95%CI 8.1–8.3), and over five years less than survivors (11.6, 95%CI 11.5–11.7). The higher level of non-employment among those who died of alcohol-related causes was mainly explained by unemployment. Among those who died of alcohol-related causes, unemployment accounted for 54% (95.5/177.5) of the total burden of non-employment, in comparison with 29% (42.9/146.0) among those who died of other causes (table 2). In contrast, the average number of days on disability pension was lower among those who died of alcohol-related causes compared to those who died of other causes. Disability pension accounted for 41% (72.4/177.5) of the total burden of non-employment among those who died of alcohol-related causes, but 65% (94.5/146.0) among those who died of other causes (table 2). No marked differences were observed in the average number of days on medically certified sick leaves (long spells) between those who died of alcohol-related causes and who died of other causes, and its contribution to total non-employment was small in both mortality groups.

**Table 2:** Sex and age adjusted average annual number of days of employment, total non-employment, unemployment, medically certified sick leave, and disability pension over the 17-year study period, among those who died of alcohol-related causes and those who died of other causes, and among survivors.

	Average annual number of days^a^		
	Alcohol-related death	Other death	Survivor
	Mean (95%CI)	Mean (95%CI)	Mean (95%CI)
**Employment**	134.9 (132.9–137.0)	175.4 (174.0–176.9)	248.5 (247. 6–249.4)
**Total non-employment**	177.5 (175.7–179.4)	146.0 (144.7–147.3)	71.1 (70.3–71.9)
Unemployment	95.9 (94.3–97.4)	42.9 (42.1–43.7)	32.2 (31.8–32.7)
Sick leave	9.3 (9.1–9.5)	8.2 (8.1–8.4)	3.8 (3.7–3.8)
Disability pension	72.4 (70.8–74.1)	94.5 (93.1–95.8)	35.2 (34.5–36.0)

a Days for employment and total non-employment do not add up to 365 days because total non-employment does not include all possible types of non-employment, and because number of days for unemployment and disability pension were extrapolated using monthly or end-of-year information.

**Figure 3:**
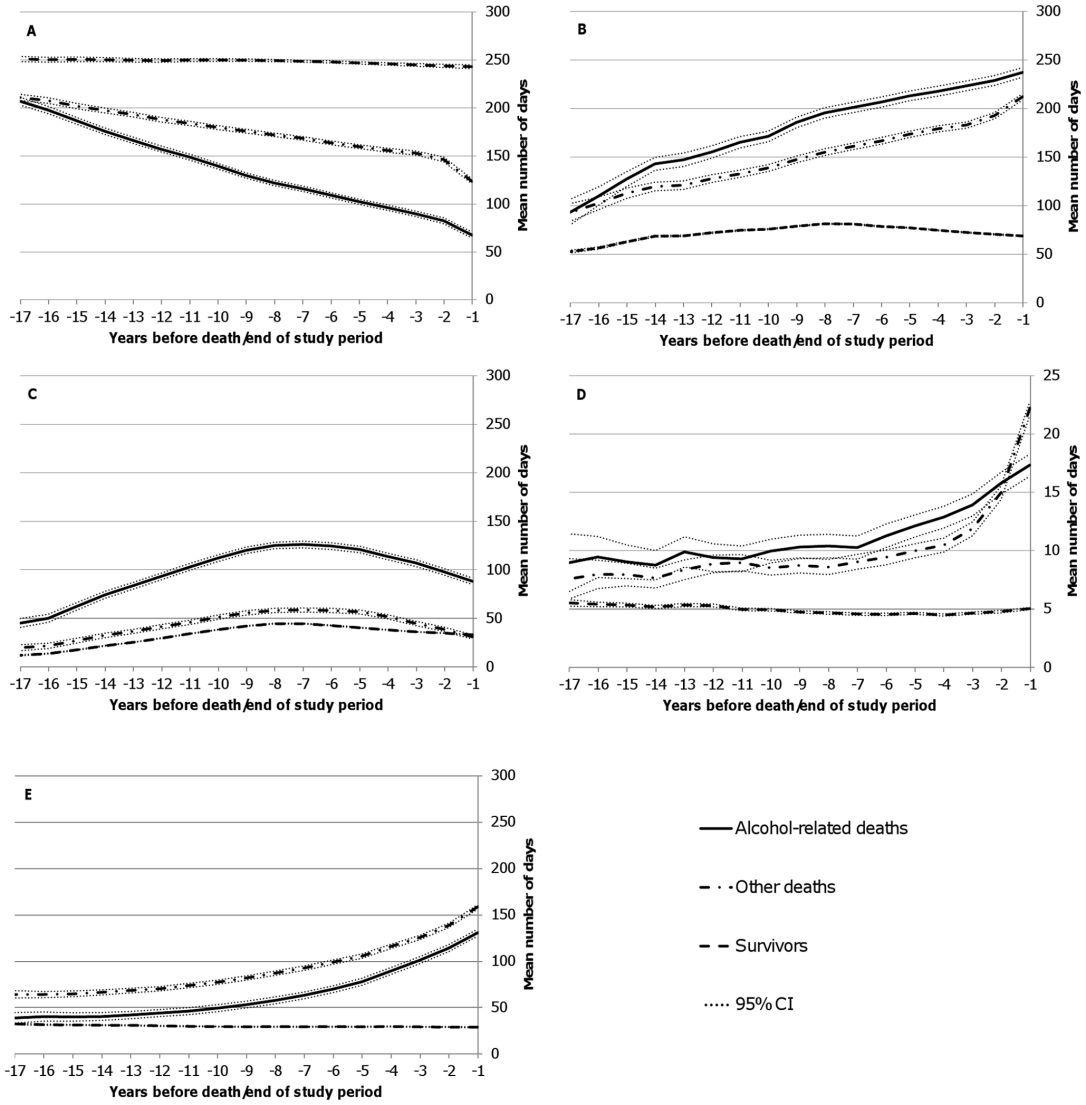
Average annual number of days of employment and non-employment. Sex and age adjusted average annual number of days of A) employment, B) total non-employment, C) unemployment, D) medically certified sick leave, and E) disability pension in the preceding 17 years before death/end of study period for those who died of alcohol-related causes, other causes, and for those who survived the 17-year period.

The employment trajectories declined already at 16 years before death in both mortality groups, but for persons dying of alcohol-related causes the trajectory decreased more steeply (figure 3A). The average rate of decline of days of employment was eight days per year among those who died from alcohol-related causes, in comparison with four days among those who died from other causes. As expected based on the employment trajectory, the total non-employment trajectory increased with the decreasing time to death in both mortality groups, and the trajectory increased more steeply among those who died of alcohol-related causes (figure 3B). The average annual number of days of unemployment was higher among those who died of alcohol-related causes already at 17 years before death compared to survivors, and to those who died of other causes (figure 3C). Taking into account the secular population trend caused by the early 1990's recession and comparing the two mortality groups, it can be seen that the level of unemployment among those who died of alcohol-related causes was disproportionally affected by the economic recession. The average rate of increase in unemployment from the beginning of the follow-up (17^th^ year before death/end of follow-up) to the peak unemployment (7^th^ year before death/end of follow-up) was eight days per year among those who died from alcohol-related causes, in comparison with four days per year among those who died from other causes. At the year when unemployment peaked (7^th^ year before death/end of follow-up), unemployment among those who died from alcohol-related causes was around two-fold higher compared to those who died from other causes and around three-fold higher compared to the survivors (general population). Relative to the unemployment level of those dying from other causes, the excess unemployment among those dying from alcohol-related causes was two-fold at the 17^th^ year before death and three-fold at the preceding year before death. The average annual number of days of medically certified sick leave did not differ to any considerable extent during the study period between the two mortality groups, with the exception of the last few years before death (figure 3D). Among those who died of other than alcohol-related causes, the average number of days on medically certified sick leave increased more steeply during the final four to three years before death compared to those who died of alcohol-related causes. The average annual number of days of disability pension was higher among those who died of other causes already at 17 years before death compared to those who died of alcohol-related causes (figure 3E). The increase in number of days of disability pension with the decreasing time to death was comparable between the two mortality groups, but the number of days on disability pension was lower among those who died of alcohol-related causes throughout the study period.

### Sensitivity analyses

The age adjusted average annual number of days of non-employment was highest among manual workers and lowest among white collar workers (table 3). This applied to all three types of non-employment, to survivors, and the two mortality groups, both among men and women.

**Table 3:** Age adjusted average annual number of days of unemployment, medically certified sick leave, and disability pension over the 17-year study period by socioeconomic status, separately for men and women.

	Men (n = 137 980)			Women (n = 120 116)		
	Alcohol-related death	Other death	Survivor	Alcohol-related death	Other death	Survivor
	Mean (95%CI)	Mean (95%CI)	Mean (95%CI)	Mean (95%CI)	Mean (95%CI)	Mean (95%CI)
**Unemployment**						
White collar worker	68.0 (64.9–71.1)	33.2 (31.7–34.6)	21.4 (20.8–22.0)	70.1 (65.9–74.3)	28.3 (27.1–29.6)	24.0 (23.6–24.5)
Manual worker	112.2 (110.1–114.3)	60.7 (59.4–62.1)	48.4 (47.6–49.2)	103.4 (98.3–108.6)	43.9 (42.0–45.8)	45.3 (44.4–46.2)
Other	65.7 (62.0–69.3)	26.1 (24.6–27.6)	24.3 (23.5–25.1)	76.7 (68.0–85.4)	23.5 (21.6–25.4)	30.8 (29.9–31.8)
**Sick leave**						
White collar worker	8.9 (8.5–9.3)	7.5 (7.2–7.7)	2.5 (2.4–2.6)	10.7 (10.0–11.3)	11.3 (11.0–11.6)	3.9 (3.8–4.0)
Manual worker	10.0 (9.7–10.2)	8.8 (8.6–9.0)	5.1 (5.0–5.3)	10.8 (10.1–11.4)	10.2 (9.9–10.5)	6.0 (5.9–6.2)
Other	9.3 (8.8–9.8)	7.6 (7.3–7.9)	3.7 (3.6–3.9)	11.0 (9.7–12.4)	8.6 (8.1–9.1)	4.4 (4.2–4.6)
**Disability pension**						
White collar worker	71.9 (68.6–75.2)	74.8 (72.4–77.2)	27.4 (26.7–28.2)	77.0 (72.0–81.9)	87.6 (85.2–89.9)	29.2 (28.6–29.9)
Manual worker	90.9 (88.8–93.0)	110.9 (109.0–112.8)	48.5 (47.6–49.5)	96.8 (91.1–102.5)	128.1 (124.7–131.4)	50.0 (48.8–51.3)
Other	80.7 (76.4–84.9)	109.8 (106.5–113.0)	42.1 (40.8–43.4)	84.0 (73.8–94.1)	137.1 (132.1–142.0)	44.0 (42.4–45.5)

We explored heterogeneity in the level of non-employment across diagnostic groups of alcohol-related causes and causes without alcohol involvement (other deaths). For this, alcohol-related causes of death (n = 15552) were divided according to the ICD–10 codes into diseases of the liver or pancreas (n = 6221; 40%), mental and behavioral disorders (n = 3577; 23%), injury due to external causes with alcohol intoxication as a contributing factor (n = 2644; 17%), alcohol poisoning (n = 2022; 13%), and other alcohol-related causes (n = 1088; 7%). Causes without alcohol involvement (n = 39166) we divided according to the ICD-10 codes into neoplasms (n = 16450; 42%), diseases of the circulatory system (n = 12925; 33%), external causes of death (n = 3916; 10%), and other diseases (n = 5875; 15%). In identifying neoplasms, diseases of the circulatory system, and external causes of death we used the following ICD-10 codes C00-D48, I00-I99, and V01-Y34, respectively.

**Figure 4:**
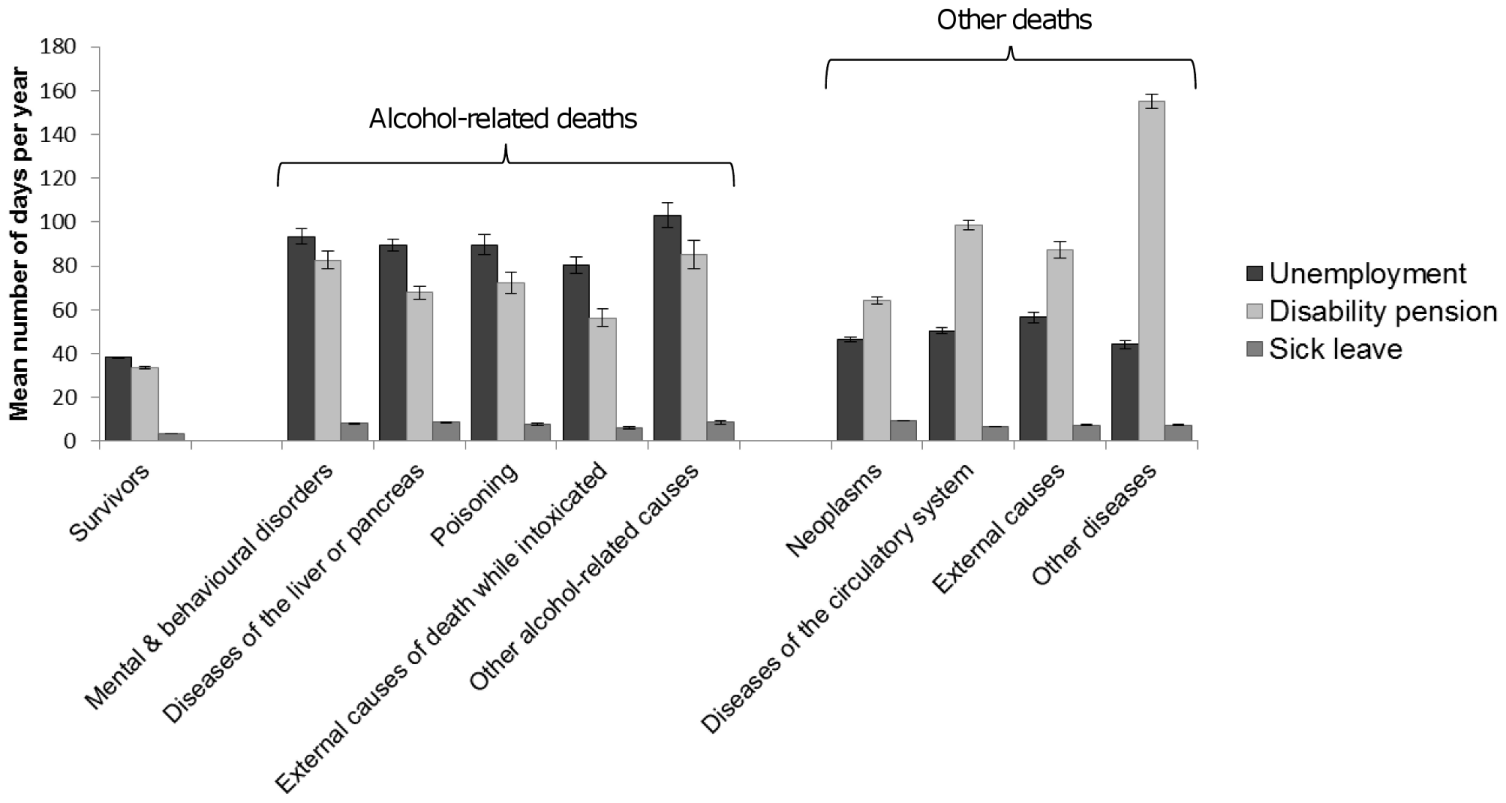
Average annual number of days of non-employment by cause of death. Sex and age adjusted average annual number of days of unemployment, disability pension, and sick leave over the 17-year study period separately by major cause-of-death groups. Error bars represent 95% confidence intervals.

Figure 4 shows the mean annual number of days of non-employment over the 17-year study period by type of non-employment for the various diagnostic sub-groups. The level of unemployment did not markedly vary within the combined cause of death groups used in the main analyses (i.e. within alcohol-related and other deaths). Therefore, the overall observation of excess unemployment among those who died of alcohol-related causes was not markedly affected by heterogeneity in cause of death sub-groups.

However, a somewhat less clear picture emerged when the overall level of disability pension was compared across the various diagnostic sub-groups than what was seen in terms of unemployment. Among those who died without alcohol involvement, the sub-group ‘other diseases’ had a markedly higher level of disability pension than any other sub-group. This cause of death sub-group consisted of diagnoses such as (chronic) diseases of the respiratory system (23%), diseases of the nervous system (20%), and diabetes mellitus (9%). The highest level of disability pension was among those whose underlying cause of death was diabetes (mean annual number of days 166.3, 95%CI 156.6–175.9). When the group of ‘other diseases’ was removed from the combined group of deaths without alcohol involvement (other deaths) the mean annual number of days of disability pension fell from 94.5 (95%CI 93.1–95.8) to 80.5 (95%CI 79.2–81.7; but was still higher than that among those who died of alcohol-related causes 72.4 (95%CI 70.8–74.1). Much (63%) of the overall excess in disability pension among those who died without alcohol involvement compared to those who died from alcohol-related causes was, therefore, explained by certain chronic conditions which can involve long-term incapacitating symptomatology, such as symptoms related to degeneration of the nervous system and chronic obstructive pulmonary disease.

Furthermore, we tested how the potential effect of the economic recession in Finland in the first half of the 1990's affected the shape of the observed unemployment trajectory. For this, we selected only those who died in 2007 and traced back their unemployment for ten years. In this analysis, which included days of unemployment only after the economic recession, the average annual number of days of unemployment showed steadily decreasing trends in both mortality groups as a function of proximity to death (models not shown). This analysis thus confirmed that the observed shape of the unemployment trajectory in figure 3C is affected by the economic recession.

## Discussion

We assessed the long-term patterning of various types of non-employment, and their relative contribution to the total burden of non-employment, in the life course of persons who experienced the most severe possible outcome of problem drinking, i.e. those who died of alcohol-related causes. We showed that the majority of these persons who died at the age of 45–64 years were in employment up to ten years before death. This means that these persons were not marginalized early on during their life course, but were actively participating in the labour market. However, they also experienced disproportionate unemployment. Compared to the level of unemployment among the general population and those who died from other than alcohol-related causes, those who died from alcohol-related causes experienced excess unemployment already at the beginning of the study period, i.e. at the 17^th^ year before death/end of follow-up. The relative contribution of the various types of non-employment differed markedly between the two mortality groups. Among those who died from alcohol-related causes, unemployment accounted for 54% of the total burden of non-employment, in comparison with 29% among those who died of other causes. Relative to the unemployment level of those dying from other causes, the excess unemployment among those dying from alcohol-related causes was two-fold at the 17^th^ year before death and three-fold at the preceding year before death.

### Methodological considerations

The strength of this study was that it was based on longitudinal information retrieved directly from various administrative registers of the institutions granting the benefits in question (for unemployment, sick leave, and disability pension). This means that we were able to reliably identify all persons who received these benefits. The 17-year retrospective study period enabled us to gain understanding of the long-term employment trajectories with the opportunity to identify potential time-dependent effects. Information on the three different types of non-employment enabled us, to our knowledge for the first time, to compare the importance of the various types of non-employment in the life course of problem drinkers who eventually died from alcohol-related causes. Comparisons with survivors (general population) and those who died from other than alcohol-related causes enabled us to identify employment histories characteristic to persons dying from alcohol-related causes. The assumption behind the comparison with those who died without alcohol involvement was that their employment trajectory represented the trajectory of those with declining health status in general. The differences between these two mortality groups can therefore highlight the special characteristics related to the typical employment trajectories of problem drinkers who eventually died from alcohol-related causes while taking into account for the effects of declining overall health status with proximity to death.

The sensitivity analyses for diagnostic sub-groups showed that in terms of unemployment, the findings were consistent. In other words, the heterogeneity in causes of death and their etiological correlates did not markedly affect the conclusions. However, in terms of disability pension the conclusions are less clear. Particularly within the group of deaths without alcohol involvement there was considerable variation in the level of disability pension by diagnostic sub-group.

Because this was a register-based study we did not have information on alcohol consumption that could have been used in determining problem drinking status. Instead, we used information on alcohol-related causes of death to identify problem drinkers. By this approach, we identified persons who had a long history of problem drinking, indicated e.g. by diagnoses of alcohol-induced liver cirrhosis and alcohol dependence. The majority of the alcohol-related diagnoses in our data were related to such diagnoses, which means that the majority were problem drinkers for a substantial time before death. We also identified persons who were problem drinkers at least at the time of death, e.g. if they died from external causes with alcohol intoxication as a contributing factor. Not all of these persons were necessarily problem drinkers for a substantial period before death, because even a single heavy drinking occasion can potentially lead to death from external causes. However, it is likely that many of those who died from external causes while intoxicated were problem drinkers earlier in their life course. This assumption is supported by a Finnish study, which showed that binge drinking predicted deaths from external causes over a 16-year period.[22] We did not have information on other health behaviours either, such as smoking. Despite the fact that many problem drinkers are known to be also heavy smokers, it does not seem plausible that problem drinkers would e.g. lose their employment mainly due to the effects of smoking and not due to problem drinking. However, the adverse synergistic effect of smoking and heavy drinking on ill health may well explain some of the observed differences in non-employment trajectories.

Not all problem drinkers die from alcohol-related causes. This means that there were problem drinkers among survivors and among those who died from causes without alcohol involvement. In relation to those who died from other than alcohol-related causes, this has probably diluted the observed differences particularly in terms of unemployment, because the level of unemployment is much higher among problem drinkers. A similar dilution effect probably applies also to comparisons with survivors, but because the group of non-problem drinkers is considerably larger than that of problem drinkers, the dilution effect on the observed trajectories is likely very limited. Furthermore, persons who die from alcohol-related causes at the age of 45–64 years are likely to suffer from a less severe progression of alcohol problems compared to those who die from alcohol-related causes at a younger age. Therefore, the employment histories of younger problem drinkers who experience severe alcohol-related harm may be even more strongly characterized by non-employment than those observed here.

In summary, because we used alcohol-related deaths as a proxy for history of problem drinking, these results probably apply only to those middle-aged problem drinkers who are at risk of experiencing similar alcohol-related health outcomes during their life course as those included in this study. Because this was a register-based study with limited information on potential confounders, the reasons for the observed differences in employment trajectories remain unclear. However, a substantial body of evidence particularly on the association between problem drinking and unemployment,[11] supports the conclusion that problem drinking and its correlates are important factors explaining the observed employment disadvantage of those who eventually died from alcohol-related causes. Future studies using prospective design should aim to replicate these findings. Given the current lack of such studies, this retrospective register-based study can provide important insights into the feasibility of preventing alcohol-related non-employment among middle-aged problem drinkers at risk of experiencing severe alcohol-related health outcomes.

### Interpretation and implications

When the non-employment trajectories were explored separately by types of non-employment (i.e. unemployment, medically certified sick leaves, and disability pensions), we found that those dying of alcohol-related conditions were markedly more likely to be unemployed at any time point during the 17-year study period before death, and among them the effect of the early 1990's economic recession on unemployment seemed to be much stronger. This finding suggests that the higher probability of unemployment among those with a severe form of problem drinking may be determined by factors that have a long-term effect during the life-course,[23], [24] and that the effect of problem drinking on the probability of being in employment is intensified during economic downturns.[11] The comparison with survivors suggests that persons dying prematurely at ages 45–64 years, for any cause, are disadvantaged in terms of unemployment; but the magnitude of the disadvantage is markedly larger and disproportionate among those dying of alcohol-related causes compared to those dying of other causes. The lower level of employment among problem drinkers who eventually died from alcohol-related causes is in line with previous research among problem drinkers without non-fatal outcomes.[5], [8], [9]

A steep increase in the annual average number of days on medically certified sick leave (periods lasting over 10 working days) during the few final years before death was seen among those dying of other than alcohol-related causes, whereas among those who died of alcohol-related causes a more modestly linearly increasing trend was observed. However, medically certified sick leaves had only a marginal role as a form of non-employment before death in both mortality groups. This can partly be explained by the fact that sickness allowance is paid approximately for one year after which, in the case of continued work disability, these persons apply for disability pension, which means that they move to other types of non-employment.

The average annual number of days on disability pension was constantly lower throughout the study period among those who died of alcohol-related causes than among those who died of other causes. The medical criteria for granting disability pension in Finland may have affected the likelihood of receiving disability benefits among these problem drinkers, because alcohol misuse per se is not accepted as the basis of disability pension. However, the lower level of disability pensions among those who died of alcohol-related causes was probably partly compensated by other non-employment pathways, such as unemployment.

Majority of risky drinkers are in employment,[25] and as we showed, also the majority of problem drinkers who eventually died of alcohol-related causes were in employment up to ten years before death. This means that workplace interventions could have an important role in reducing alcohol-related premature exit from employment. The feasibility of workplace alcohol screening and brief intervention is supported by its expected positive impact e.g. on employee productivity,[26] and by the fact that alcohol screening seems to be widely accepted by the public.[27] However, while the effectiveness of alcohol screening and brief intervention has been consistently shown in health care settings, effectiveness studies in workplace settings are almost non-existent.[28], [29]

One key challenge in preventing alcohol-related exit from labour force is that the perceived need for treatment among persons with alcohol use disorders (AUD) is low, and thus most individuals with an AUD are not in treatment or actively seek treatment.[30], [31] Those with more severe AUD symptoms may be more likely to perceive a need for treatment than others,[32] but among these persons markedly progressed ill health may affect their ability to gain and maintain employment.[33] Persons with AUDs are also frequently disadvantaged in terms of recovery capital meaning that these persons often lack various personal and social resources needed in overcoming substance abuse disorders.[17], [34] Furthermore, accumulation of factors potentially producing synergistic adverse effects among persons with AUDs, such as living alone and being unemployed,[35] also contribute to the increased risk of experiencing alcohol-related harm. Also in our data the majority of problem drinkers who eventually died from alcohol-related causes were manual workers and non-married. However, based on their long-term employment histories, our results suggest that even the majority of these persons are employable, provided that they are identified and treated early enough.

## Conclusions

Our results add to the understanding of the relation between a severe form of problem drinking and non-employment by showing that the majority of persons who died from alcohol-related causes were in employment up to ten years before death. Therefore, based on their long-term employment history, these persons should not be seen as ‘hopeless cases’,[20] but instead they represent a group of persons who can be part of the productive workforce, provided that they are identified for prevention early enough during their working career. However, their disproportionate movement to unemployment is problematic from the point of view of prevention because utilization of health care services is known to be low among unemployed despite their various unmet health needs.[36], [37] The results, therefore, also suggest that integrating alcohol screening, brief intervention, and referral to treatment as part of employment services could prove to be effective in preventing alcohol-related permanent exit from the labour force.
